# Exploring plant growth promoting traits and biocontrol potential of new isolated *Bacillus subtilis* BS-2301 strain in suppressing *Sclerotinia sclerotiorum* through various mechanisms

**DOI:** 10.3389/fpls.2024.1444328

**Published:** 2024-08-22

**Authors:** Muhammad Ayaz, Qurban Ali, Wei Zhao, Yuan-Kai Chi, Farman Ali, Khan Abdur Rashid, Shun Cao, Yan-qiu He, Abdul Aziz Bukero, Wen-Kun Huang, Ren-De Qi

**Affiliations:** ^1^ Institute of Plant Protection and Agro-Products Safety, Anhui Academy of Agricultural Sciences, Hefei, China; ^2^ State Key Laboratory for Biology of Plant Diseases and Insect Pests, Institute of Plant Protection, Chinese Academy of Agricultural Sciences, Beijing, China; ^3^ Department of Biology, College of Science, United Arab Emirates University, Al-Ain, Abu-Dhabi, United Arab Emirates; ^4^ Department of Entomology, Abdul Wali Khan University, Mardan, Pakistan; ^5^ Department of Plant Pathology, Key Laboratory of Monitoring and Management of Crop Diseases and Pest Insects, College of Plant Protection, Ministry of Education, Nanjing Agricultural University, Nanjing, China; ^6^ MARA-CABI Joint Laboratory for Bio-safety, Institute of Plant Protection, Chinese Academy of Agricultural Science, Beijing, China

**Keywords:** *Bacillus subtilis*, stem white mold, growth promotion, ROS, oxalic acid

## Abstract

*Sclerotinia sclerotiorum* (Lib.) de Bary is the causative agent of stem white mold disease which severely reduces major crop productivity including soybean and rapeseed worldwide. The current study aimed to explore plant growth-promoting traits and biocontrol of new isolated *Bacillus subtilis* BS-2301 to suppress *S. sclerotiorum* through various mechanisms. The results indicated that the BS-2301 exhibited strong biocontrol potential against *S. sclerotiorum* up to 74% both in dual culture and partition plate experiments. The BS-2301 and its crude extract significantly suppressed *S. sclerotiorum* growth involving excessive reactive oxygen species (ROS) production in mycelia for rapid death. Furthermore, the treated hyphae produced low oxalic acid (OA), a crucial pathogenicity factor of *S. sclerotiorum*. The SEM and TEM microscopy of *S. sclerotiorum* showed severe damage in terms of cell wall, cell membrane breakage, cytoplasm displacement, and organelles disintegration compared to control. The pathogenicity of *S. sclerotiorum* exposed to BS-2301 had less disease progression potential on soybean leaves in the detached leaf assay experiment. Remarkably, the strain also demonstrated broad-range antagonistic activity with 70%, and 68% inhibition rates against *Phytophthora sojae* and *Fusarium oxysporum*, respectively. Furthermore, the strain exhibits multiple plant growth-promoting and disease-prevention traits, including the production of indole-3-acetic acid (IAA), siderophores, amylases, cellulases and proteases as well as harboring calcium phosphate decomposition activity. In comparison to the control, the BS-2301 also showed great potential for enhancing soybean seedlings growth for different parameters, including shoot length 31.23%, root length 29.87%, total fresh weight 33.45%, and total dry weight 27.56%. The antioxidant enzymes like CAT, POD, SOD and APX under BS-2301 treatment were up-regulated in *S. sclerotiorum* infected plants along with the positive regulation of defense-related genes (*PR1-2*, *PR10, PAL1*, *AOS*, *CHS*, and *PDF1.2)*. These findings demonstrate that the BS-2301 strain possesses a notable broad-spectrum biocontrol potential against different phytopathogens and provides new insight in suppressing *S. sclerotiorum* through various mechanisms. Therefore, BS-2301 will be helpful in the development of biofertilizers for sustainable agricultural practices.

## 1 Introduction

White stem mold, caused by the devastating fungus *Sclerotinia sclerotiorum*, globally affects major crop productivity. The pathogen is highly notorious, affecting nearly 500 plant species, including rapeseed, sunflower, cabbage, soybean, beans, and carrot. Studies on *S. sclerotiorum-*infected plants show reduced growth, leaf lesions, and wilting, which can lead to plant death (Massawe et al., 2018; Farzand et al., 2019a). The development of sclerotia and mycelial growth has been identified as pathogenicity factors in *S. sclerotiorum*, but oxalic acid (OA) is mainly considered a major influencer of pathogenicity in plants (Massawe et al., 2018; Farzand et al., 2019a). Polygalacturonases, pectinolytic cell wall-degrading enzymes (CWDE), have also been identified as important pathogenicity factors (Smolińska and Kowalska, 2018; Farzand et al., 2019a). These enzymes become active when the OA establishes a low pH environment (pH 3-4). A mutant of *S. sclerotiorum* lacking OA exhibits less pathogenicity against susceptible plants, based on previous studies (Liang et al., 2015; Massawe et al., 2018). *S. sclerotiorum* produces sclerotia to survive in harsh environments for long time, but under favorable conditions, it germinate to form apothecia containing ascospores and spread the disease (Foley et al., 2016; Taylor et al., 2018).

Many control strategies, such as breeding resistant crops, moisture monitoring, and crop rotation are considered less effective for managing *S. sclerotiorum* due to sclerotia formation in the soil and its potential to infect multiple crops. Applying synthetic fungicides is the most effective control strategy (Jones et al., 2014; Zhai et al., 2023). However, the use of excessive chemicals has raised serious issues regarding environmental pollution and human health. Recently, researchers have shown more interest in biological control as a successful strategy to manage plant diseases without negatively impacting the environment (Ayaz et al., 2021). It has been shown that biological control agents (BCAs), particularly *Bacillus* endophytes, can protect plants from multiple phytopathogens such as fungi, bacteria, and nematodes (Fira et al., 2018; Ayaz et al., 2023). Many plant-associated microorganisms have been screened for their ability to suppress phytopathogens both *in vitro* and *in plant* experiments, providing direction for novel BCAs identification. To date, the well-known antagonistic bacteria identified from the rhizosphere of various plants are *Pseudomonas* and *Bacillus* species (Chen et al., 2009; Wang et al., 2018).

*Bacillus* spp. produces a wide range of compounds that promote plant growth, promotion, and control diseases. They can regulate phytohormones and defense-related genes during biotic stress (Wu et al., 2019; Gu et al., 2022). *Bacillus* spp. suppress pathogens by regulating plant antioxidant enzymes, such as superoxide dismutase (SOD), catalases (CAT), peroxidase (POD), phenylalanine ammonia-lyase (PAL), ascorbate peroxidase (APX) and polyphenol oxidase (PPO) (Xu et al., 2008; Rais et al., 2017). Studies have shown positive regulation of defense-related genes, such as *PR1-2*, *PR10*, *CHS*, *PAL1*, and *PDF1.2* in soybean plants infected with pathogens under plant growth promoting rhizobacteria treatment (Xu et al., 2012; Zhai et al., 2023). *Bacillus* spp. can also stimulate plant growth by solubilizing soil nutrients and nitrogen fixation. For example, *B. velezensis* FZB42 enhances tomato growth by regulating phytohormone production, nutrient absorption, and root development under biotic stress (Borriss, 2011; Wu et al., 2014). It was also observed that *Bacillus* spp. in the rhizosphere effectively colonizes roots for biofilm formation to control *Ralstonia solanacearum* (Chen et al., 2013). *Bacillus* spp. can produce resistant endospores and a wide range of antimicrobial compounds. Introducing novel *Bacillus* strains in biocontrol strategies could reduce the need for chemical fertilizers and support sustainable agriculture (Xu et al., 2008; Wang et al., 2018).

The agriculture industry could greatly benefit from the discovery of novel BCAs. The objective of the current study is to examine the plant growth-promoting traits and biocontrol potential of the newly isolated *B. Subtilis* BS-2301 in soybean plants, with a focus on its ability to suppress *S. sclerotiorum* through various mechanisms. The BS-2301 exhibited broad-spectrum antagonistic activity and effectively suppressed *S. sclerotiorum* by reducing sclerotia formation, decreasing oxalic acid production, inducing excessive ROS production, and causing structural deformities in the pathogen hyphae. Additionally, the strain was found to regulate antioxidant enzymes, plant growth, and defense-related genes in infected soybean plants. The present work provides new insights related to the biocontrol potential of the BS-2301 strain using various mechanisms to combat *S. sclerotiorum*. These findings suggest that BS-2301, with its strong biocontrol potential against *S. sclerotiorum*, could be utilized in different formulations to develop biopesticides for sustainably managing white mold disease in agriculture sector.

## 2 Material and methods

### 2.1 Strain identification, plant pathogens, and growth conditions

The BS-2301, isolated from the rhizosphere of a soybean plant was provided by Dr. Zhao Wei in the Institute of Plant Protection and Agro-product Safety, Anhui Academy of Agricultural Sciences, Hefei (31° 50’ 54.2328’’ N and 117° 16’ 20.5032’’ E) China. All fungal and oomycetes plant pathogens, such as *S. sclerotiorum, Fusarium oxysporum*, and *Phytophthora sojae*, were supplied by Prof. Dr. Rende Qi from the same institute. The strain BS-2301 and DH5α were grown in Luria Bertani (LB) broth overnight at 25°C and stored in a 60% (v/v) glycerol solution at -80°C for future use (Ayaz et al., 2022). The fungal pathogens were maintained on PDA, while the Oomycetes phytopathogens were grown in V8 juice at 25°C for 5-7 days to obtain fresh cultures (Farzand et al., 2019a; Yu et al., 2023). The BS-2301 strain was identified as a *B. subtilis* based on 16S rRNA sequencing. Universal primers 27F (5’-AGAGTTTGATCMTGGCTCAG-3′) and 1492R (5’-TACGGYTACCTTGTTACGACTT-3′) were used to amplify the 16S rRNA of BS-2301 (Jo et al., 2020). A Basic Local Alignment Tool (BLAST) for the 16S rRNA sequence was performed to determine its similarity with closely related bacterial strains (Ki et al., 2009). Subsequently, the strain was sent to “Novogene, Beijing, China” for whole genome sequencing. The local cultivar of soybean (Zheng 1307) seed was surface serialized for 3-5 minutes with 5% sodium hypochlorite and 70% ethanol solutions, respectively, then rinsed with ddH_2_O thrice. The seeds were air-dried on a clean bench before being used for *in vitro* and *in planta* experiments (Zubair et al., 2019).

### 2.2 Plant growth promoting traits

The newly isolated BS-2301 was evaluated for plant growth-promoting traits, including IAA production, phosphate solubilization, siderophore production, and biofilm formation described below.

#### 2.2.1 Indole acetic acid production

Briefly, the Salkowski method was used to detect IAA production by BS-2301. After inoculating 5 μL of a fresh overnight culture of BS-2301 into 5 mL of LB medium supplemented with 2.5 mg/mL of tryptophan, the mixture was cultured for 7 days at 28°C. Subsequently, 2 mL of the supernatant was collected and mixed with 4 mL of Salkowski reagent (12 g/L of FeCl_3_ and 8 M H_2_SO_4_) and incubated for 30 minutes at room temperature in the dark. The presence of pink color in the BS-2301 sample indicated IAA production (Khan et al., 2023).

#### 2.2.2 Siderophores production

The BS-2301 strain was used for siderophore production on chromeazurol-S (CAS) agar medium. The first 5 μL of fresh overnight BS-2301 culture was spotted on the CAS agar plate for siderophore production. After 7 days of incubation at 28°C, the appearance of an orange translucent zone indicated positive activity in siderophore formation by the strain (Xu et al., 2019).

#### 2.2.3 Biofilm formation

The strain was also analyzed for its potential to form a stable biofilm following a previous protocol (Zhai et al., 2023). The bacterial cells from overnight culture 1×10^8^ CFU mL^−1^ were collected and washed thrice with sterilized water. Subsequently, 1 mL of fresh LB medium and 100 μL of the bacterial culture were added to a 24-well polystyrene plate (Jiang et al., 2013). As a control, a bacteria-free sterilized LB medium was used. The plate was then incubated for 12, 24, 48, 72, 96, and 120 hours at 28°C. After incubation the developed biofilm was stained for 20 minutes using a 0.1% crystal violet aqueous solution. It was then rinsed three times with sterile distilled water and instantly decolorized for 20 minutes using a 1 mL solution of 95% ethanol. The crystal violet absorbance was measured at OD_570_ to evaluate the biofilm formation by BS-2301 (Peeters et al., 2008). Additionally, 2 mL of fresh LB medium was added to a polystyrene 6-well plate with sterile 1 cm cover glasses. Afterward, 20 μL (1×10^8^ CFU mL^−1^) of BS-2301 bacterial suspension was added to the LB medium and then stained with crystal violet. The cover glass was rinsed off the unbound dye solution and left to air dry. The biofilm developed on the glass cover was examined under an optical microscope with 10x and 40x magnifications at various time intervals. The experiment was performed thrice with at least five replicates (Zhai et al., 2023).

#### 2.2.4 Phosphate solubilization assay

The phosphate solubilization activity was screened for BS-2301 on LB media supplemented with Ca3(PO4)2 (Pi medium) following the previous protocol (Chen and Liu, 2019). The BS-2301 capacity to solubilize phosphate was determined by the formation of clear zones surrounding the colony.

### 2.3 Extracellular enzymes production

The strain was also screened for extracellular enzymes such as proteases, cellulases, and amylases using previously published protocols.

#### 2.3.1 Proteases activity

Briefly, to assess the protease activity, the BS-2301 was incubated at 28°C for 4 days on Nutrient Agar (NA) enriched medium with 1% skim milk. The presence of transparent, white-background zones surrounding the colony indicated proteolytic activity (Artha et al., 2019).

#### 2.3.2 Amylase activity

For amylase activity, the BS-2301 strain was grown on an NA medium supplemented with 1% starch and incubated at 28°C for 4 days. After that, Lugol’s iodine solution (1%) was added to Petri plates, and the development of clear zones around the colony demonstrated the presence of amylase activity (Artha et al., 2019).

#### 2.3.3 Cellulases activity

The medium supplemented with CMC was used for the cellulase enzyme production activity assay. The BS-2301 strain was inoculated on a CMC-enriched NA medium. To detect cellulolytic activity, the Petri plates were covered with congo red solution. Clear zones from pink to yellow around the BS-3201 colony, indicated the presence of cellulase enzyme activity (Artha et al., 2019).

### 2.4 Dual culture and partition petri plates antagonistic assay

A dual-culture and partition plate assays were carried out to observe the inhibition potential of BS-2301 against fungal and oomycete pathogens directly or indirectly (Massawe et al., 2018; Farzand et al., 2019a). In brief, for the direct antagonistic assay, small plugs (0.5 cm) of the selected pathogens (*S. Sclerotiorum*, *F. oxysporum*, and *P. sojae*) were placed in the center of each petri plate filled with PDA or V8 media. Subsequently, on a sterilized filter paper disc, 3 cm away from the pathogen plug, 5 µL of BS-2301 overnight culture (10^6^ CFU mL^-1^) and its crude extract (15µL) were inoculated. After sealing the Petri plates with parafilm, they were incubated for 4-6 days at 28°C (Farzand et al., 2019a). Using the partition plate approach, the antifungal efficacy of BS-2301 was investigated to prevent direct contact with the tested pathogens (Massawe et al., 2018). Briefly, 0.5 cm paper discs were impregnated with 5 μL of the overnight BS-2301 culture (10^6^ CFU mL^-1^) on one side of the partition plate containing LB media. Subsequently, a 0.5 cm diameter plug of each pathogen was placed on the other side with PDA or V8 media required for fungal and oomycetes pathogens respectively. The parafilm-sealed plates were incubated at 28°C for up to 6 days. The pathogen’s mycelium growth inhibition (%) was calculated using the formula below:


Rate of Inhibition (%)= [(C−T)/C]×100

where C and T represent the mycelial growth length of each pathogen in the control (without BS-2301) and treatment (inoculated with BS-2301) from the center to the edges of the plate, respectively. The experiments were conducted four times, with at least five replicates for each treatment.

### 2.5 The impact of BS-2301 on sclerotia amount, development, and viability

The sclerotia assay was conducted using the dual-culture method. Briefly, *S. sclerotiorum* mycelial plugs (0.5 cm) from a fresh culture were sliced and placed on a PDA in the middle of a plate. The BS-2301 overnight culture (10^6^ CFU mL^-1^) or its crude extract (15 µL) was impregnated on a paper disc 3 cm away from the fungal plugs. The plates were then tightly sealed with parafilm and incubated at 25°C. The number, mean weight, texture, and interior color of the sclerotia were visually examined at 14 days post-inoculation (dpi) under different treatments (Massawe et al., 2018).

### 2.6 Assessment of reactive oxygen species

Excessive production of ROS in the hyphae causes severe damage to the fungus, leading to rapid death. ROS accumulation was evaluated in *S. sclerotiorum* mycelia treated with BS-2301 using fluorescence microscopy and the probe dichloro-dihydro-fluorescein diacetate (DCFH-DA) (Ayaz et al., 2021). Untreated hyphae were used as a control. Briefly, *S. sclerotiorum* was cultured at 25°C for 96 hours and exposed to BS-2301 and its crude extract. The treated mycelia were collected and transferred to 10 mM sodium phosphate buffer (pH 7.4) into Eppendorf tubes (1.5 mL). Subsequently, 10 μL of DCFH-DA (Solarbio^®^ CAT No: D6470) was added to the samples for staining and incubated at 25°C in the dark for 30 minutes. The DCHF-DA stained damaged mycelia produced green fluorescence were examined using an image-pro express software (6.2) Olympus 1 x 71 microscope (Olympus, Tokyo, Japan).

### 2.7 Morphological and ultrastructural changes in *S. sclerotiorum* mycelia

The damage in *S. sclerotiorum* mycelia was observed using scanning electron microscopy (SEM) and transmission electron microscopy (TEM) under different treatments compared with control. The collected mycelia were washed three times in a 100 mM phosphate buffer after being treated with 2.5% glutaraldehyde to prepare them for SEM. Subsequently, the samples were dehydrated using an ethanol gradient and post-fixed for three hours in an osmium tetroxide (1%) solution. After that, the samples were coated on gold particles and analyzed at a 5 kV voltage using a Hitachi S-3000 N SEM (Hitachi). The prefixed samples were divided into pieces with an ultra-microtome, placed inside an Epon 812 for TEM analysis, and observed under a Hitachi H-600 TEM (Farzand et al., 2019a; Ali et al., 2023).

### 2.8 Oxalic acid production assay in BS-2301 treated *S. sclerotiorum* mycelia

*S. sclerotiorum* was assessed for its oxalic acid production in PDA media supplemented with bromophenol, an indicator of oxalic acid production. To study the impact of BS-2301 and its crude extract on oxalic acid production of *S. sclerotiorum* during the antagonistic assay, fungal plugs (0.6 cm) were sliced from the edge of the inhibitory zone and placed on the bromophenol blue-modified PDA plates (Massawe et al., 2018). These plates were then incubated at 25°C for 96 hours. The width of the yellow acidification zone, indicating OA production, was measured at 24-hour intervals following a previously established protocol (Massawe et al., 2018; Farzand et al., 2019a). The experiment was repeated five times, with three replicates for each treatment.

### 2.9 Biocontrol efficacy of BS-2301 against *S. sclerotiorum* on detached leaves

To evaluate the biocontrol efficacy of BS-2301 against *Sclerotinia* stem rot on detached leaves, a local soybean cultivar (Zheng 1307) susceptible to the disease was used. Leaves from 30-day-old rapeseed and soybean plants were surface sterilized using 75% ethanol for 3 minutes, rinsed three times in sterile distilled water, and air-dried on sterile filter paper. *S. sclerotiorum* mycelial plugs (0.6 cm) from the edge of the inhibition zones were excised and inoculated on detached leaves. Untreated *S. sclerotiorum* served as the control (not exposed to BS-2301) (Cao et al., 2023). Additionally, in partition plates, detached leaves with healthy fungal plugs (0.6 cm) were exposed to BS-2301 volatiles grown in the second compartment on the LB medium. The sealed parafilm plates were kept at 25°C, 85% RH an 8-h light and 16-h dark photoperiod for 5 days. Lesion sizes were measured to assess the biocontrol efficacy of different BS-2301 treatments against *S. sclerotiorum* on detached leaves (Massawe et al., 2018). The experiment was repeated five times, with three replicates of each treatment.

### 2.10 Plant defense enzymes and malondialdehyde analysis

Soybean seedlings (V2 stage) were inoculated with BS-2301 overnight fresh culture (10^8^ CFU mL^-1^), with H_2_O used as a control (Zhai et al., 2023). After 72 h of inoculation, *S. sclerotiorum* mycelial plugs (0.6 cm) were placed on the stem of each plant, which had been previously wounded with a sterilized razor. Leaves samples were collected at 4 dpi for analysis of defense enzymes. The activity of four key antioxidant enzymes such as ascorbate peroxidase (APX), superoxide dismutase (SOD), catalase (CAT), and peroxidase (POD) was determined using kits (Nanjing Jiancheng Bioengineering Institute) protocols (Yong et al., 2008). Briefly, frozen leaf samples (0.3 g) were crushed in a phosphate buffer solution (pH 7.8) containing 1 mM EDTA over an ice bath. The resulting mixture was centrifuged at 1200 × g for 30 minutes at 4 °C, and the supernatant was collected as the final enzyme extract. The absorbance activity of SOD, APX, CAT, and POD was measured at 450, 290, 405, and 420 nm respectively, with ddH_2_O serving as a reference. The malondialdehyde (MDA) was determined following the protocol previously reported (Ali et al., 2023).

### 2.11 Growth promotion and induction of systemic resistance in soybean plants by BS-2301

*In vitro* experiments were conducted to observe the direct and indirect effects of the BS-2301 on soybean seedling growth (Ali et al., 2023; Khan et al., 2023). Briefly, surface sterilization for 5 minutes of soybean seeds was carried out with sterilized sodium hypochlorite (5%) followed by ethanol (70%) solutions, then washed 4 times with sterilized water (ddH_2_O) and placed in an overnight culture of BS-2301 (10^8^ CFU mL^-1^) for 15 minutes. After air drying on a clean bench, the seeds were transferred to sterilized 9 cm Petri plates containing 0.8% MS medium. For the indirect effect, the partition plates approach was utilized to observe the impact of BS-2301-VOCs on soybean growth promotion. The partition plate was set up with three soybean seeds on one side and LB agar medium having BS-2301 on opposite side were kept for 12 days at 25°C under 16 h light/8 h dark photoperiod in a growth chamber.

A Pot experiment was conducted to validate the *in vitro* findings, demonstrating BS-2301 role in enhancing plant growth and disease suppression. Soybean seeds were surface sterilized with 70% ethanol and 5% sodium hypochlorite solutions, followed by rinsing with ddH_2_O. The effectiveness of the sterilization process was confirmed by incubating LB plates with the wash water at 37°C for 4 days which showed no bacterial growth. Three soybean seeds were grown in plastic glasses (6 x 3 cm) containing sterilized soil and at the V2 stage the plants were inoculated with an overnight culture of BS-2301 (10^8^ CFU mL^-1^). After 72 h, *S*. *sclerotiorum* plugs were placed on the soybean stems, which were previously wounded and wrapped with parafilm. Disease severity was assessed at 10 dpi by measuring lesion size compared to the control (Farzand et al., 2019a; Zhai et al., 2023). The experiment was repeated thrice with at least four replicates for each treatment.

### 2.12 Expression profiling of defense-related genes

The expression profiling of defense-linked genes (*PR1-2*, *PR10, PAL1*, *AOS*, *CHS*, and *PDF1.2*) in soybean plants was evaluated in a pot experiment. The experiment included four treatments (і) soybean plants treated with water (CK), (i) soybean plants treated with BS-2301 only, (ii) soybean plants inoculated with *S. sclerotiorum* (S.s), and (iii) soybean plants containing both BS-2301 and *S. sclerotiorum* (S.s+BS-2301) following a previously reported protocol (Zhai et al., 2023). Leaves from different treatments were collected 96 h after *S. sclerotiorum* inoculation. Leaf samples were used for total RNA extraction using the Trelief ™ RNAprep pure Plant kit (Lot Number: TSP411, China) and cDNA synthesis was performed using the Goldenstar™ RT6 cDNA synthesis Kit ver.2 (Beijing TsingKe Biotech Co., Ltd). Gene sequences (**Supplementary Table S1**) were obtained from NCBI on January 15, 2024, and primers were designed using the Primer Quest online application (https://sg.idtdna.com/PrimerQuest/Home/Index). The housekeeping actin-3 (Gene ID=100798052) was used as a reference. The selected gene expression was determined using a Bio-RAD thermal cycler (CFX96TM Real-time system, USA) with the following PCR program: 30 seconds of initial denaturation at 95°C, 40 cycles at 95°C for 5 seconds, and 34 s at 60°C. Finally, 2^-ΔΔCt^ C comparative was exploited for relative quantification (Zubair et al., 2019).

### 2.13 Statistical analysis

Each experiment was carried out with a completely randomized design. Standard deviations (SD) were used to express the results based on at least four replications (n = 4). An analysis of variance (ANOVA) was performed, followed by the Tukey Honestly Significant Difference (HSD) to determine significant differences at p ≤ 0.05. The experimental data were analyzed using Statistix 8.1 software, and the graphics were generated using Origin’s graphics and analysis program (Version 2022, Origin Lab Corporation).

## 3 Results

### 3.1 BS-2301 genetic features and plant growth promoting traits

**Figure 1:**
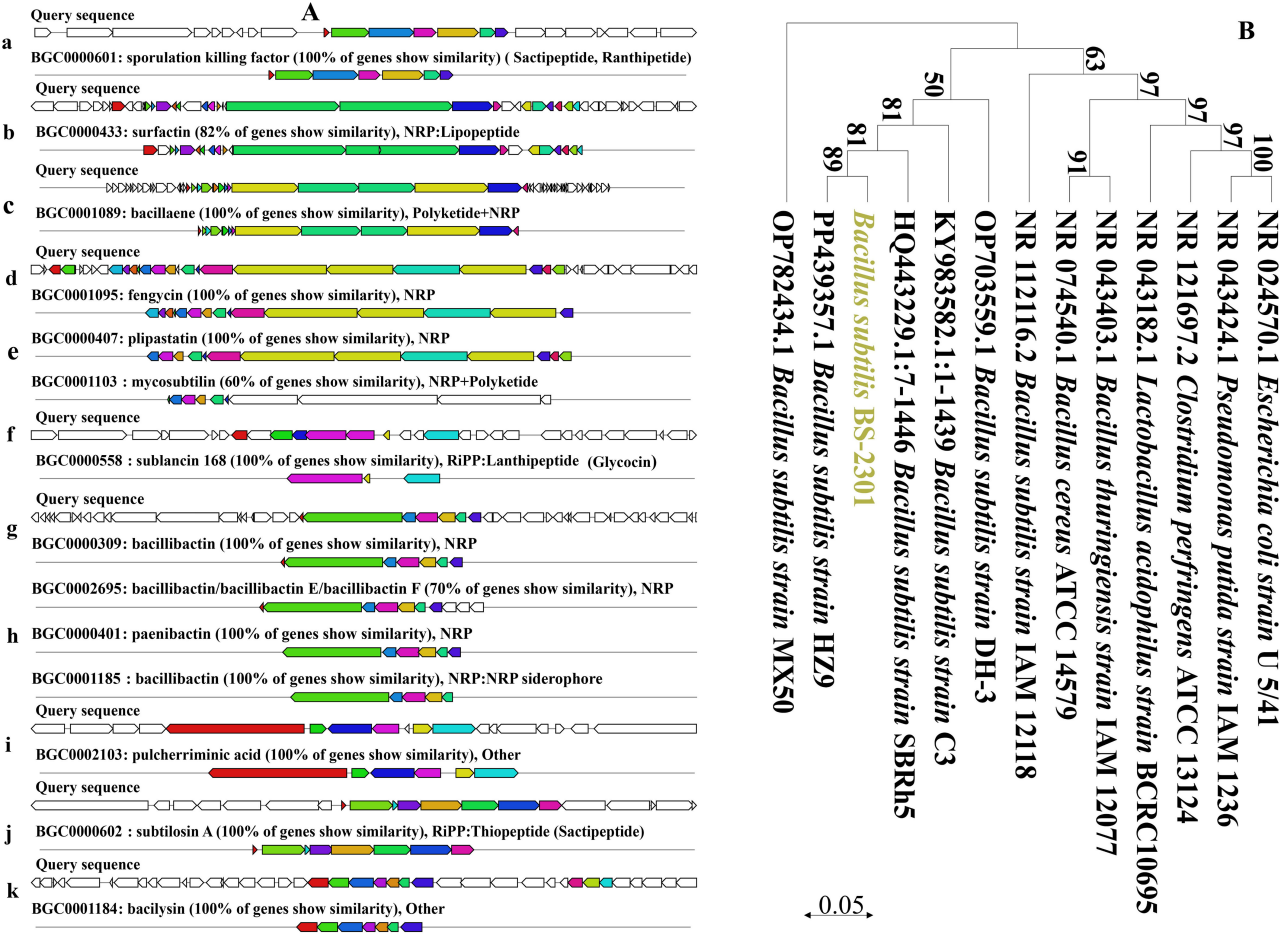
Detail of various gene clusters prediction resposible for antimicrobial compounds synthesis in BS-2301 genome and phylogenetic analysis of BS-2301 based on 16S rRNA **(A)** Prediction of gene clusters for diffeent antimicrobial compounds synthesis **(a-k)** in BS-2301 genome through antiSMASH bacterial version online tool **(B)** Phylogenetic tree conntructed from 16S rRNA using neighbour joining method comaparison with closely related Bacterail strains. The phylogentic tree is made through MEGA 11 software and the bootstrap value are mentioned at each node point.

**Figure 2:**
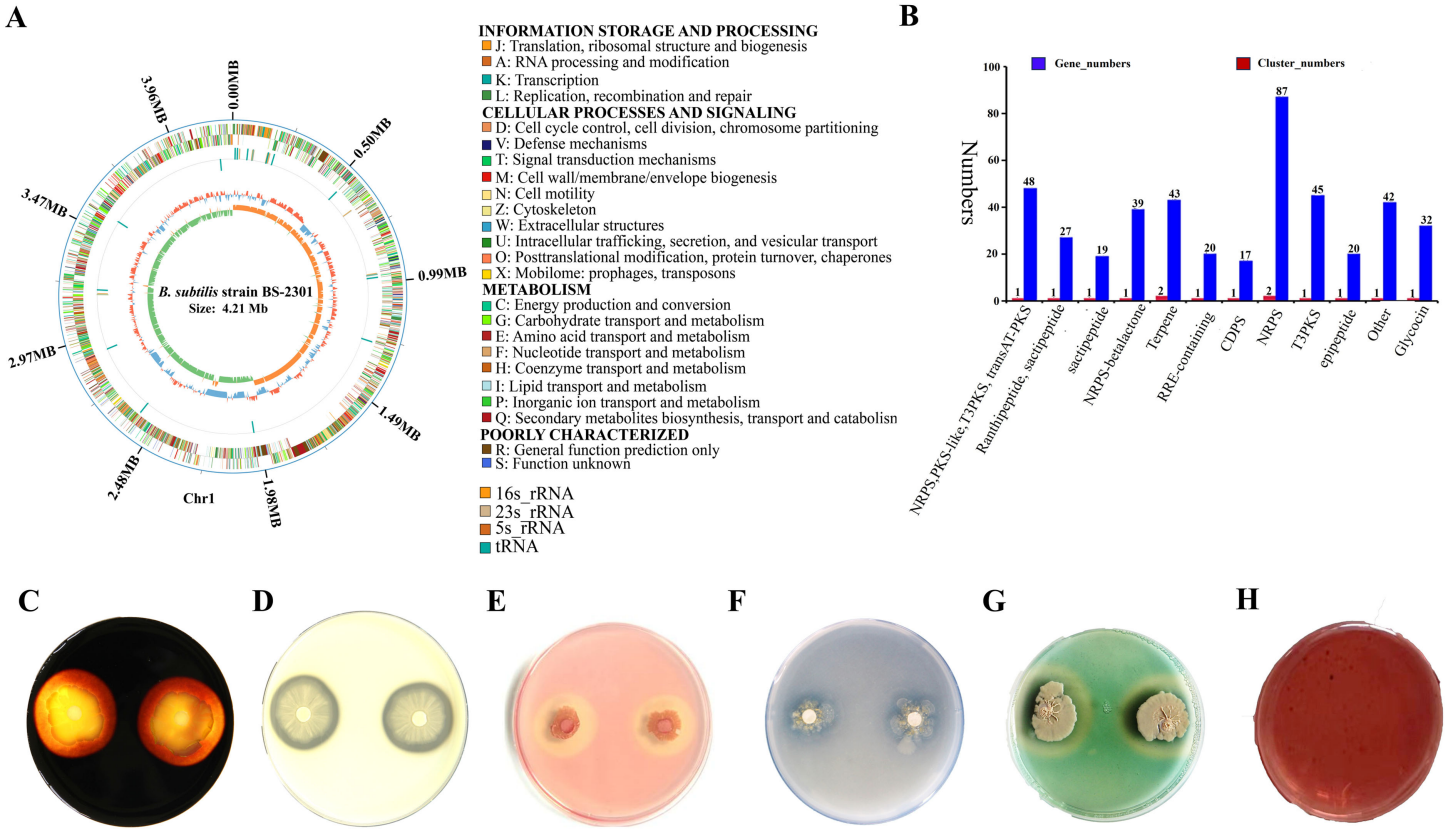
Genetic features and plant growth-promoting traits of *B*. *subtilis* strain BS-2301. **(A)** BS-2301 circular chromosome map in GC view and COG function classification **(B)** Secondary metabolites gene clusters **(C)** amylase **(D)** protease **(E)** cellulase **(F)** phosphate solubilization **(G)** siderophore production and **(H)** Indole Acetic Acid (IAA) assay. Each experiment was repeated thrice with five replicates.

Based on 16S rRNA and whole-genome sequencing data, the newly isolated strain belongs to *B. subtilis*. The 16S rRNA sequence of BS-2301 closely matched with *B. subtilis* (**Figure 1B**) using NCBI blast analysis. The 16S rRNA of BS-2301 is submitted to GenBank (Accession: PQ013083). The strain was further sequenced using PacBio Sequel II and Illumina NovaSeq PE150 platforms, resulting in a 4.21 Mb circular chromosome with 43.51% GC content. The total genome contains 4,674 predicted genes grouped into different categories. Anti-smash software analysis revealed 14 secondary metabolite clusters (**Figure 1A**) including surfactin, fengycin, plipastatin, bacillaene, sactipetide, ranthipeptide, bacilysin and bacillibactin. BS-2301 exhibits various plant growth-promoting traits, involving the production of IAA for plant growth promotion. The strain also produces extracellular enzymes, such as cellulase, protease, amylase, and siderophore that mostly play an important role in the biocontrol of phytopathogens and plant growth promotion. Inoculation of BS-2301 on the agar medium showed positive halos for siderophore, cellulase, protease, and amylase production. Additionally, BS-2301 exhibited phosphate solubilization ability, as indicated by a clear halo zone on Pikovskaya (PVK) agar medium (**Figure 2**).

### 3.2 Broad spectrum biocontrol potential against different phytopathogens

**Figure 3:**
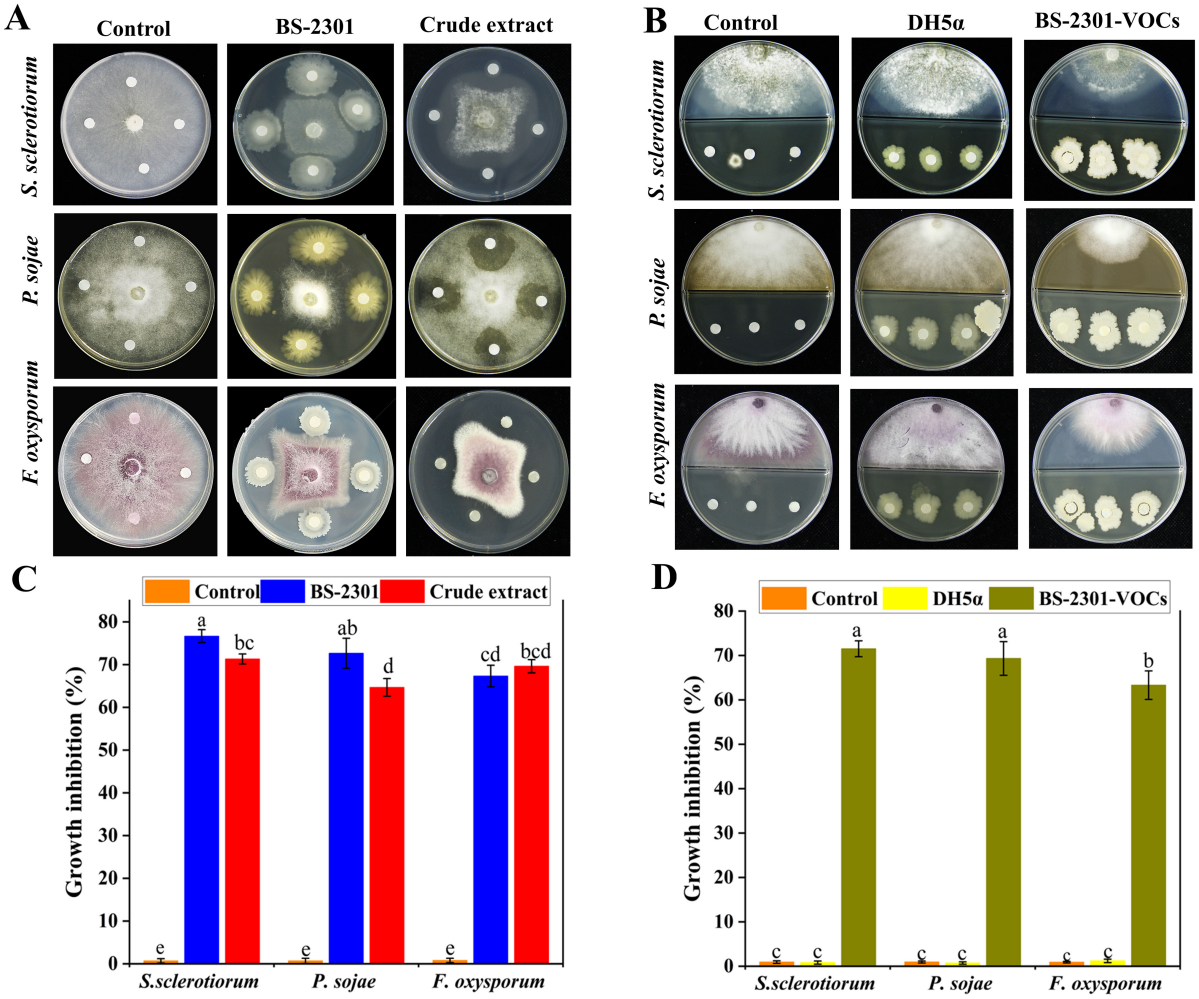
Broad spectrum antagonistic potential of BS-2301 against *S. sclerotiorum*, *P. sojae*, and *F*. *oxysporum.* **(A)** Dual culture method was used to study the direct effect of BS-2301 and crude extract on the selected pathogens compared to the control **(B)** Partition Petri plate experiment showing the indirect inhibitory effect of BS-2301-VOCs on above-mentioned phytopathogens compared to DH5α and control **(C)** Graphical representation of BS-2301 direct effect on growth inhibition of selected pathogens **(D)** Graphical display of BS-2301-VOCs antagonistic effect on above pathogens. Each experiment was repeated thrice with five replicates. The lowercase letters on the columns show significant differences among the treatments using Tukey’s HSD test.

The broad-spectrum biocontrol potential of BS-2301 against various phytopathogens, including *S. sclerotium*, *P. sojae*, and *F. oxysporum* were observed in the present work. The results indicated that BS-2301 and its crude extract showed significant antagonistic activity against *S. sclerotium*, followed by *P. sojae* and *F. oxysporum*, with growth inhibition rates of 74%, 70%, and 68%, respectively. The antagonistic effect of BS-2301 varied among the selected phytopathogens, ranging from 68% to 75%, with the highest inhibition observed against *S. sclerotiorum* (**Figures 3A, C**).

Furthermore BS-2301-VOCs, demonstrated strong broad-spectrum antagonistic activity against the selected pathogens in partition Petri plate experiments compared to DH5α and the control. BS-2301-VOCs were most effective against *S. sclerotium*, followed by *P. sojae* and *F. oxysporum*, with growth inhibition rates of 72%, 67%, and 65%, respectively (**Figures 3B, D**). Overall, the findings suggest that BS-2301 possesses strong broad-spectrum biocontrol potential, with the highest inhibitory effect observed against *S. sclerotiorum* in both dual culture and partition plate experiments.

### 3.3 Evaluation of oxidative damage in *S. sclerotiurum* mycelia

**Figure 4:**
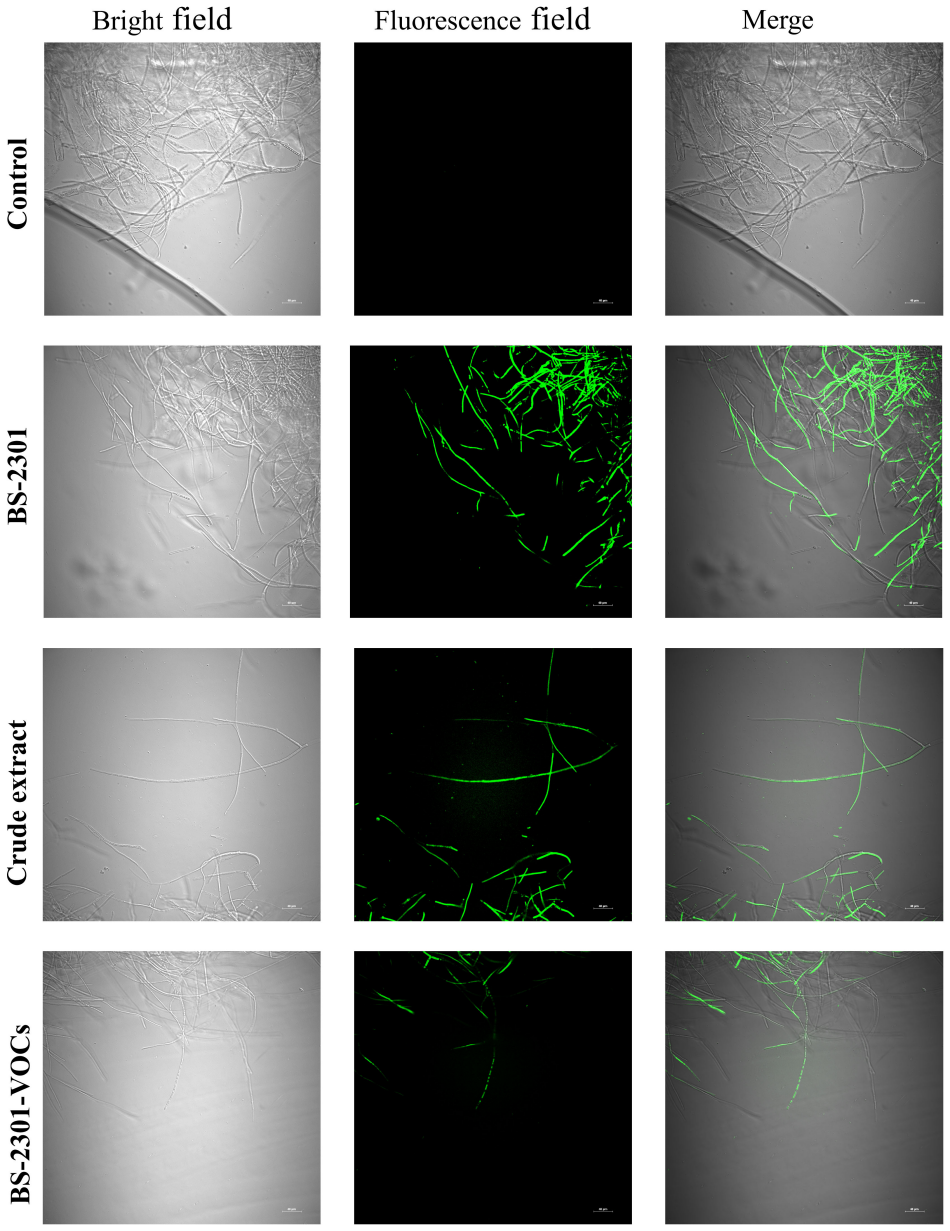
Formation of Reactive oxygen species (ROS) using bright and green field microscopy in *S. sclerotiorum* mycelia treated with BS-2301, crude extract, and BS-2301-VOCs. Using DCFH-DA staining, ROS in the mycelia after different treatments was observed. The scale bar represents 40 μm.

*S. sclerotiorum* hyphae exposed to BS-3201, crude extract, and BS-2301-VOCs accumulated ROS that led to oxidative stress and rapid cell death. The results indicated that BS-2301 and crude extract significantly induced oxidative damage in the treated mycelia compared to BS-2301-VOCs (**Figure 4**). The untreated control plates showed no damage to hyphae under green and white light fluorescence. Overall, the findings demonstrated that BS-2301 in the dual culture method significantly increased oxidative stress in *S. sclerotiorum*, followed by the crude extract and BS-2301-VOCs.

### 3.4 Reduction in *S. sclerotiorum* disease progression on soybean detached leaves

**Figure 5:**
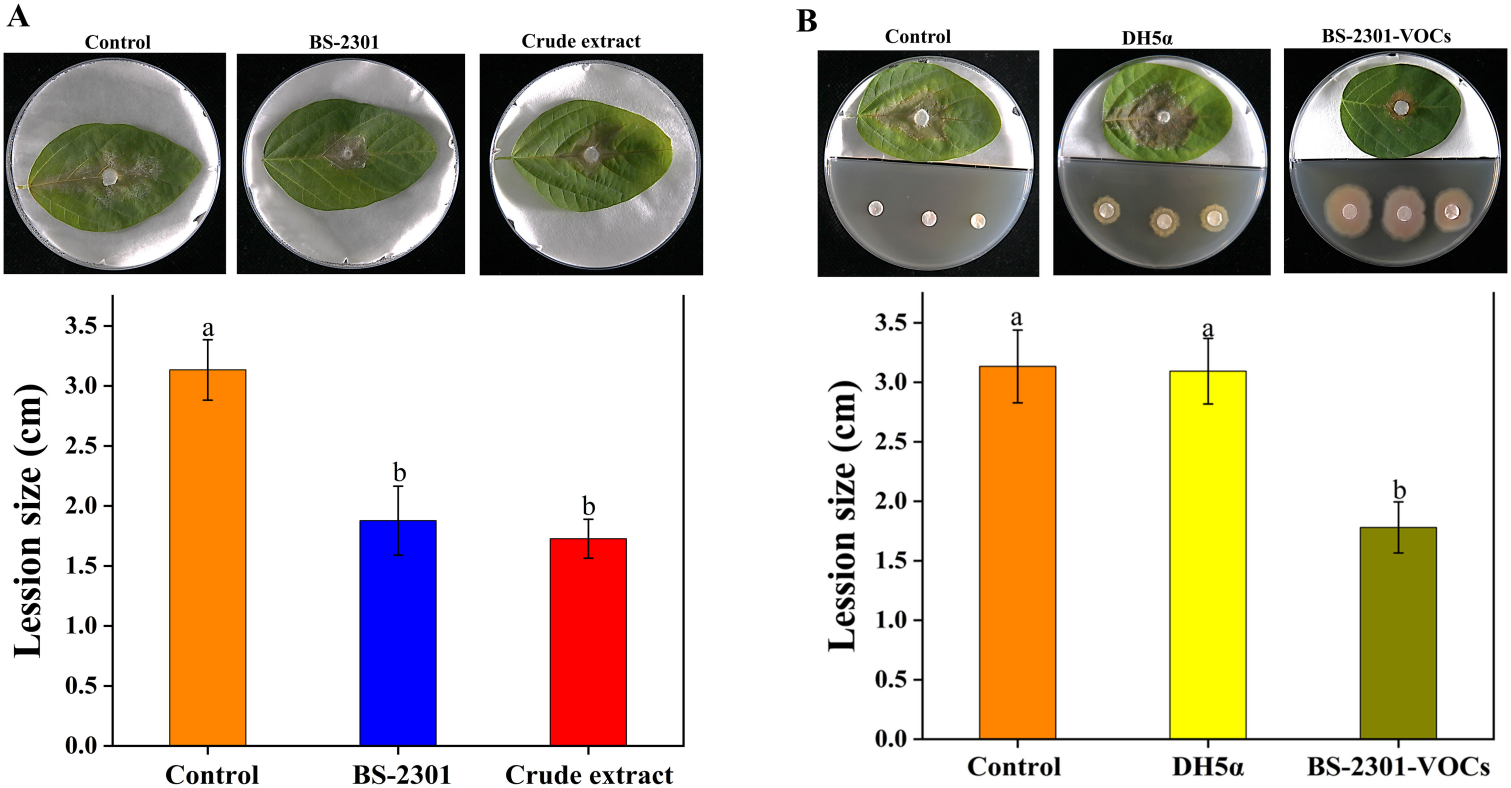
Detached leaf assay for analyzing the biocontrol efficacy of BS-2301, crude extract, and BS-2301-VOCs against *S. sclerotiorum* disease on soybean leaves. **(A)** Reduction in *S.sclerotiorum* disease progression treated with BS-2301 and crude extract compared to control (healthy fungal plugs) on soybean leaves **(B)** The indirect effect of BS-2301-VOCs on *S. sclerotiorum* disease progression on soybean leaves compared to DH5α and control. The experiment was repeated three times with five replicates for each treatment. The lowercase letters above the columns indicate significant differences among the treatments, following Tukey’s HSD test.

The efficacy of BS-2301 in controlling *S. sclerotiorum* was evaluated using a soybean detached leaf assay. Results showed a significant decrease in disease severity on soybean leaves treated with BS-2301 and crude extract compared to control. The lesion diameter on leaves treated with BS-2301 and crude extract was notably smaller than on control leaves inoculated with healthy fungal plugs. The control group exhibited more severe symptoms, with an average lesion diameter of 3.2 cm, while BS-2301 and crude extract groups had diameters of 1.6 cm and 1.9 cm, respectively (**Figure 5A**). Furthermore, the indirect impact of BS-2301-VOCs on S. *sclerotiorum* progression indicated a significant reduction in disease progression on soybean leaves compared to DH5α and the control (**Figure 5B**). Overall BS-2301, crude extract, and BS-2301-VOCs effectively inhibited *S. sclerotiorum* disease progression on soybean leaves compared to the control and DH5α.

### 3.5 *S. sclerotiorum* oxalic acid production, sclerotia number, and mycelia morphology

**Figure 6:**
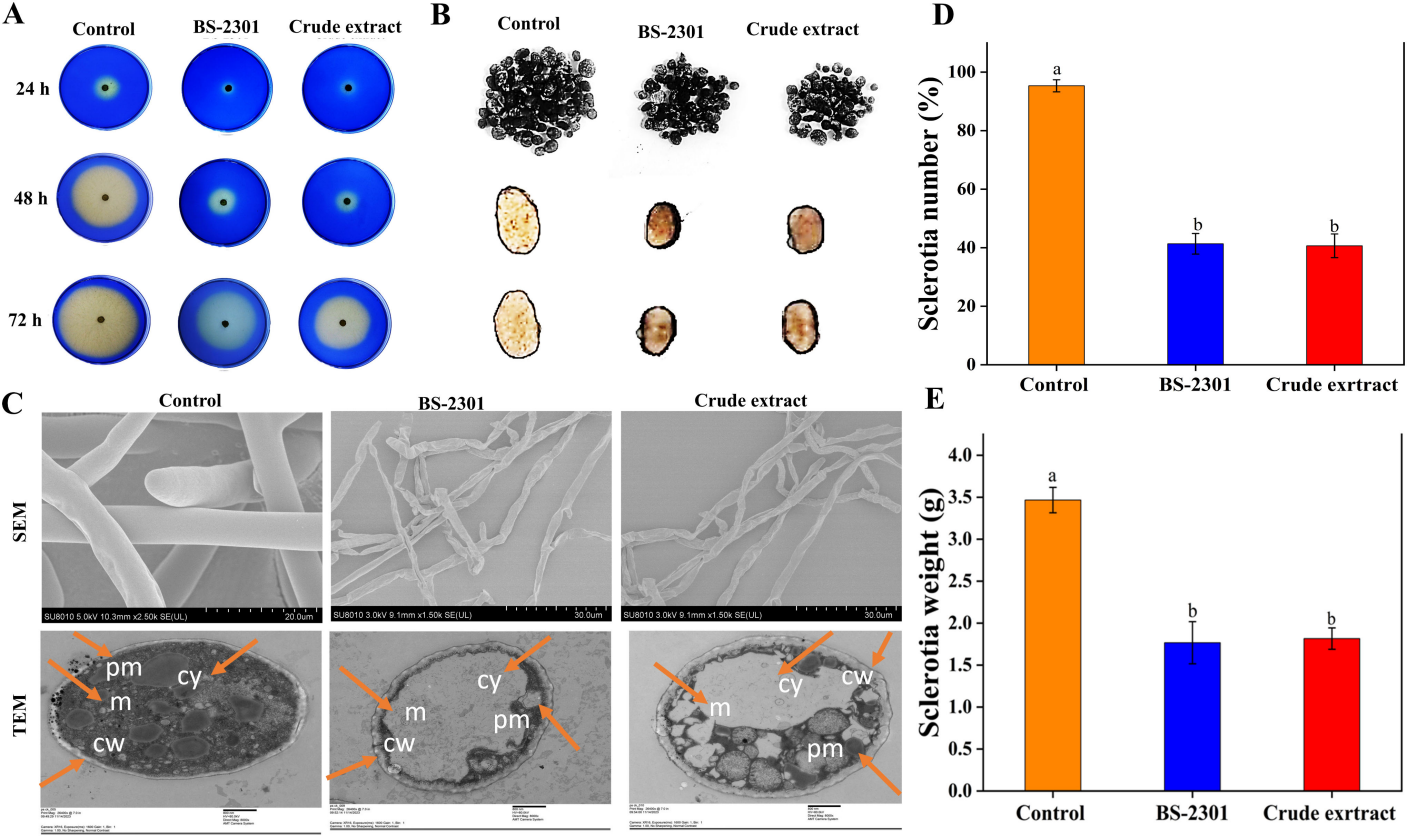
The effect of BS-2301 and crude extract on *S. sclerotiorum* oxalic acid production, sclerotia formation, and mycelial morphology **(A)** Illustration of the bromophenol blue acidification assay for oxalic acid production in treated and untreated *S. sclerotiorum* **(B)** Illustration of sclerotia formation in *S. sclerotiorum* under BS-2301 and crude extract treatments **(C)** Examination of treated *S. sclerotium* under SEM and TEM microscopy (cy, cytoplasm, pm, plama membrane, cw, cell wall and m, mitochondria) **(D)** Graph showing the number of sclerotia **(E)** Graph showing the weight of sclerotia. The experiment was repeated three times with five replicates for each treatment. The lowercase letters above the columns indicate significant differences among the treatments, following Tukey’s HSD test.

Various experiments, including the OA production assay, sclerotia number assessment, and examination of mycelial morphological and ultrastructural changes, were conducted to evaluate the detrimental effects of BS-2301 and its crude extract on *S. sclerotiorum*. The results of the bromophenol blue acidification assay showed that BS-3201 and the crude extract significantly reduced OA production of *S. sclerotiorum* compared to the control (untreated fungal plugs). Healthy untreated fungal plugs exhibited yellow coloration at 48 and 72 hpi and grew faster than those treated with BS-2301 and the crude extract. Treated fungal plugs failed to acidify the medium at 72 hpi, indicated by the retention of blue color compared to the control. Some slight acidification was observed in BS-2301 and crude extract-treated fungal plates at 72 hpi, along with reduced mycelial growth (**Figure 6A**).

Following the observed inhibition of *S. sclerotiorum* mycelial growth, the impact of BS-3201 and the crude extract on sclerotia production and viability was investigated. The results revealed a significant decrease in sclerotia formation in treated *S. sclerotiorum* compared to the control. The number and weight of sclerotia were reduced by up to 50% in both BS-2301 and crude extract-treated fungal plates, with smaller and softer sclerotia formed in the treated groups (**Figures 6B, D, E**).

Morphological and ultrastructural changes in *S. sclerotiorum* mycelia were examined using SEM and TEM. Control hyphae appeared long, dense, and cylindrical under SEM, while BS2301 and crude extract-treated fungal hyphae displayed deformities, such as plasmolysis, curling, shrinkage, pore formation, and distortion. TEM analysis breakdown of the cell wall and membrane, loss of cellular integrity, cell shrinkage, membrane damage, uneven cell thickness, displacement of cellular contents, and leakage of cytoplasmic material in BS-2301 and crude extract-treated *S. sclerotiorum*, contrasting with the intact structure of untreated fungal mycelia. (**Figure 6C**).

### 3.6 Biofilm formation by BS-2301

**Figure 7:**
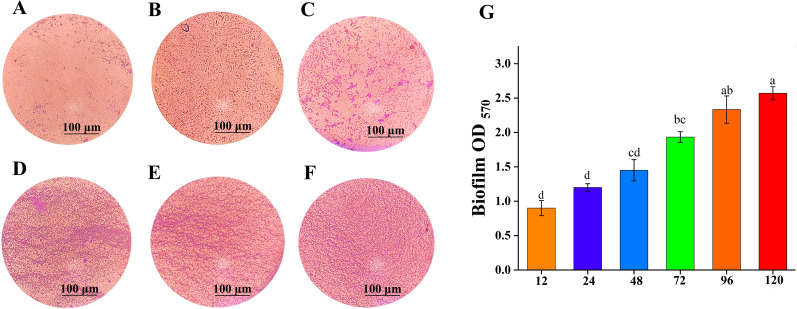
Biofilm formation ability of BS-2301 at various time intervals. **(A–F)** shows biofilm formation stained with crystal violet dye at 12, 24, 48, 72, 96, and 120 hours, respectively. **(G)** Biofilm formation optical density at OD_570_ at different time intervals. The lowercase letters above the columns indicate significant differences among the treatments based on Tukey’s HSD test.

The ability of BS-2301 to produce biofilm was determined under different time intervals using an optical microscope (**Figures 7A–F**). The results showed that BS-2301 cells were dispersed and had low adhesion before 24 h. After 48 h, some cells began to cluster and form distinctive biofilm structures. Over time, the number of bacteria adhering to the cover glass increased significantly, and reached at peak after 120 hours. Biofilm formation was quantified using an ultraviolet spectrophotometer at OD_570_ (**Figure 7G**), showed a steady increase in biofilm production over time, with the highest levels observed after 120 h

### 3.7 *In vitro* plant growth promotion in soybean seedlings by BS-2301

**Figure 8:**
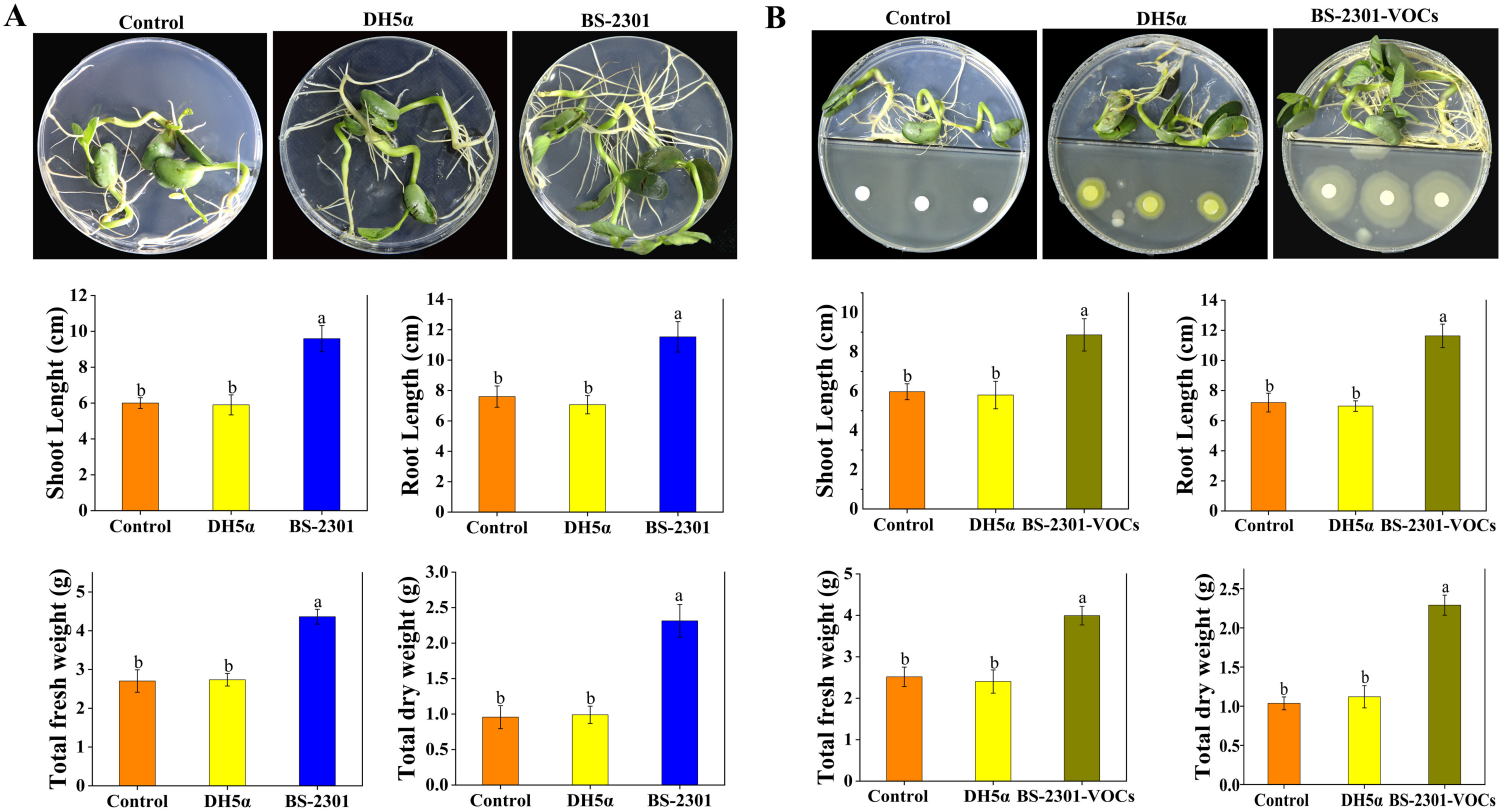
*In vitro* Petri plates experiments showing the direct and indirect effect of BS-2301 and BS-2301-VOCs on soybean seedling growth. **(A)** Comparison of soybean seedling growth directly influenced by BS-2301, DH5α, and control. **(B)** Visual presentation of the impact of BS-2301-VOCs on soybean seedlings’ growth compared to DH5α and the control. The graphs show the measurements of shoot length, root length, total fresh weight, and total dry weight for both direct and indirect experiments. The lowercase letters above the columns represent significant differences among the treatments based on Tukey’s HSD test at P ≤ 0.05.

Soybean seeds inoculated with BS-2301 exhibited higher germination rates and increased seedling growth compared to DH5α and the control (**Figure 8A**). The findings demonstrated that both direct application of BS-2301 and indirect exposure to BS-2301-VOCs enhanced soybean seedling growth leading to increased shoot length (up to 35%), root length (up to 29%), total fresh weight (up to 33%) and total dry weight (up to 28%). Additionally, it was observed that the direct impact of BS-2301 on soybean seedling growth was slightly greater than the indirect effect of BS-2301-VOCs (**Figure 8B**).

### 3.8 *In planta* biocontrol efficacy of BS-2301 against *S. sclerotiorum* in soybean plants

**Figure 9:**
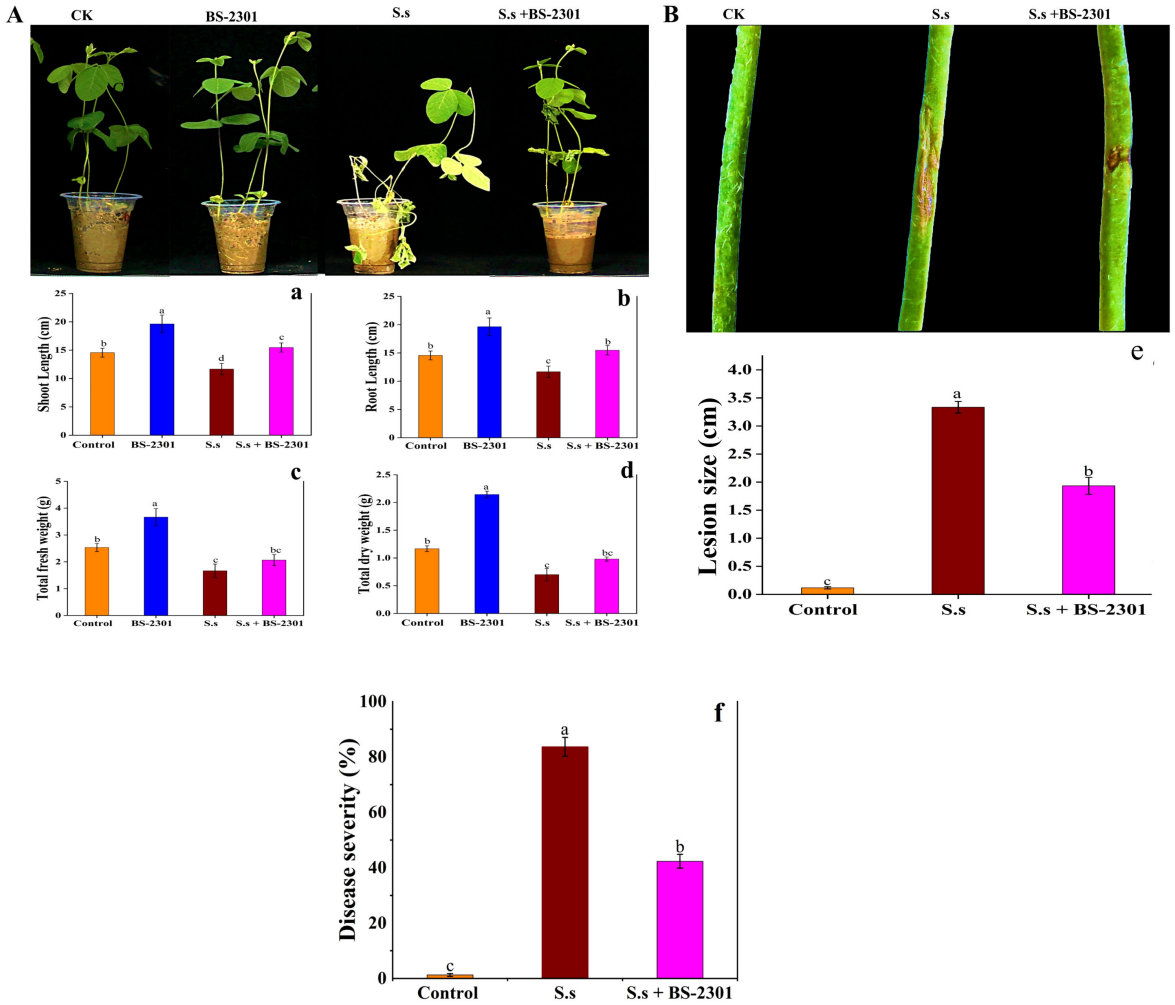
Effect of BS-2301 on soybean growth promotion and reduction of *S. sclerotiorum* disease. **(A)** Illustration of BS-2301 soybean growth promotion under different treatments **(B)** visual representation of reduction in *S. sclerotiorum* lesions on soybean under BS-2301 treatment. Graphical representation of shoot length (a), root length (b), total fresh weight (c), and total dry weight (d), graph showing *S. sclerotiorum* lesion size on soybean stems under different treatments (e), disease severity (f). The lowercase letters on the columns show significant differences among treatments, following Tukey’s HSD test at P ≤ 0.05.

Pot experiment showed that BS-2301 significantly enhanced soybean growth and reduced disease severity caused by *S. sclerotiorum* in soybean plants. Soybean plants inoculated with BS-2301 exhibited maximum growth compared to the control group (**Figure 9A**). BS-2301 treatment led to increased shoot length, root length, total fresh weight, and total dry weight in soybean plants, including those with *S. sclerotiorum* plugs on the stem (S.s + BS-2301) (**Figure 9A**). The growth promotion rate under BS-2301 treatment were 37% for shoot length, 30% for root length, 33% for total fresh weight, and 28% for total dry weight. The results indicated significant growth promotion in healthy soybean plants treated with BS-2301, followed by those with *S. sclerotiorum* (S.s + BS-2301) as shown in **Figure 9A**. Plants with *S. sclerotiorum* plugs on the stem without BS-2301 treatment (S.s) exhibited larger lesion sizes and high disease severity while BS-2301-treated soybean plants (S.s + BS-2301) exhibited reduced disesse severity and small lesion szie on stem (**Figure 9B**). These findings demonstrate the strong biocontrol efficacy of BS-2301 against *S. sclerotiorum* disease progression in soybean plants.

### 3.9 Defence enzymes regulation by BS-2301

**Figure 10:**
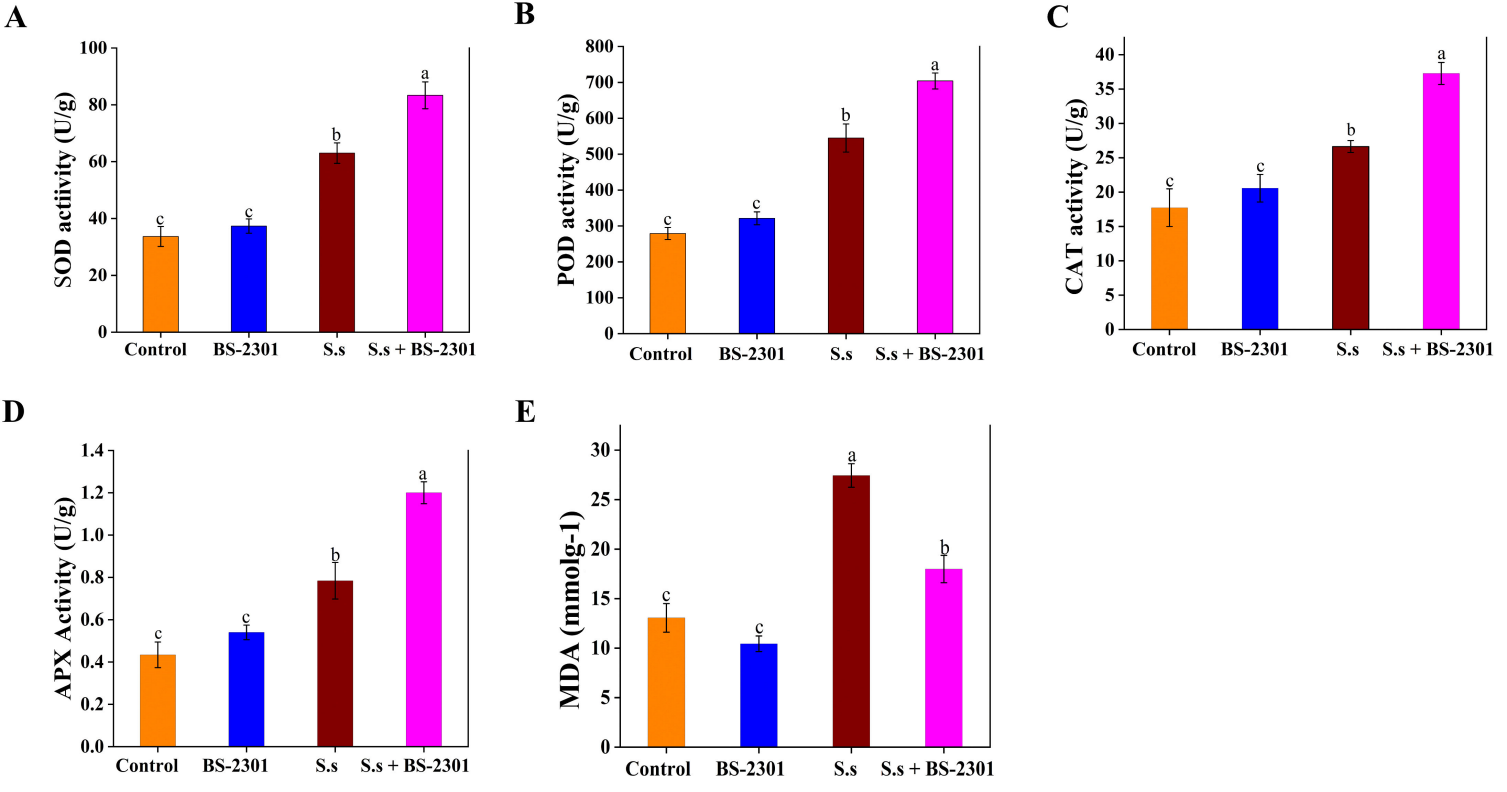
Regulation of defense enzymes and MDA levels in soybean under BS-2310 treatment. **(A)** Catalaze (CAT), **(B)** Superoxidase dismutase (SOD), **(C)**, Peroxidase (POD), **(D)** Ascorbate peroxidase (APX), and **(E)** Malondialdehyde (MDA). The significant difference among treatments was indicated by lowercase letters above the columns through Tuckey’s HSD test at P ≤ 0.05.

Compared to plants infected with *S. sclerotiorum* alone, soybean plants treated with BS-2301 showed a significant increase in the activity of plant defense enzymes. Specifically, SOD activity increased by 34%, CAT by 30%, POD by 27%, and APX by 25% (**Figures 10A–D**). These findings suggest that BS-2301 may regulate defense enzymes in soybean plants to reduce the excessive oxidative stress caused by *S. sclerotiorum* infection. The results also showed a significant reduction (35%) in MDA levels in infected plants treated with BS-2301 (S.s + BS-2301), indicating a reduction in oxidative stress (**Figure 10E**). Overall, the findings demonstrate that BS-2301 plays a crucial role in regulating soybean defense enzymes to mitigate oxidative stress in *S. sclerotiorum*-infected plants.

### 3.10 Profiling of defense-related genes in soybean plants

**Figure 11:**
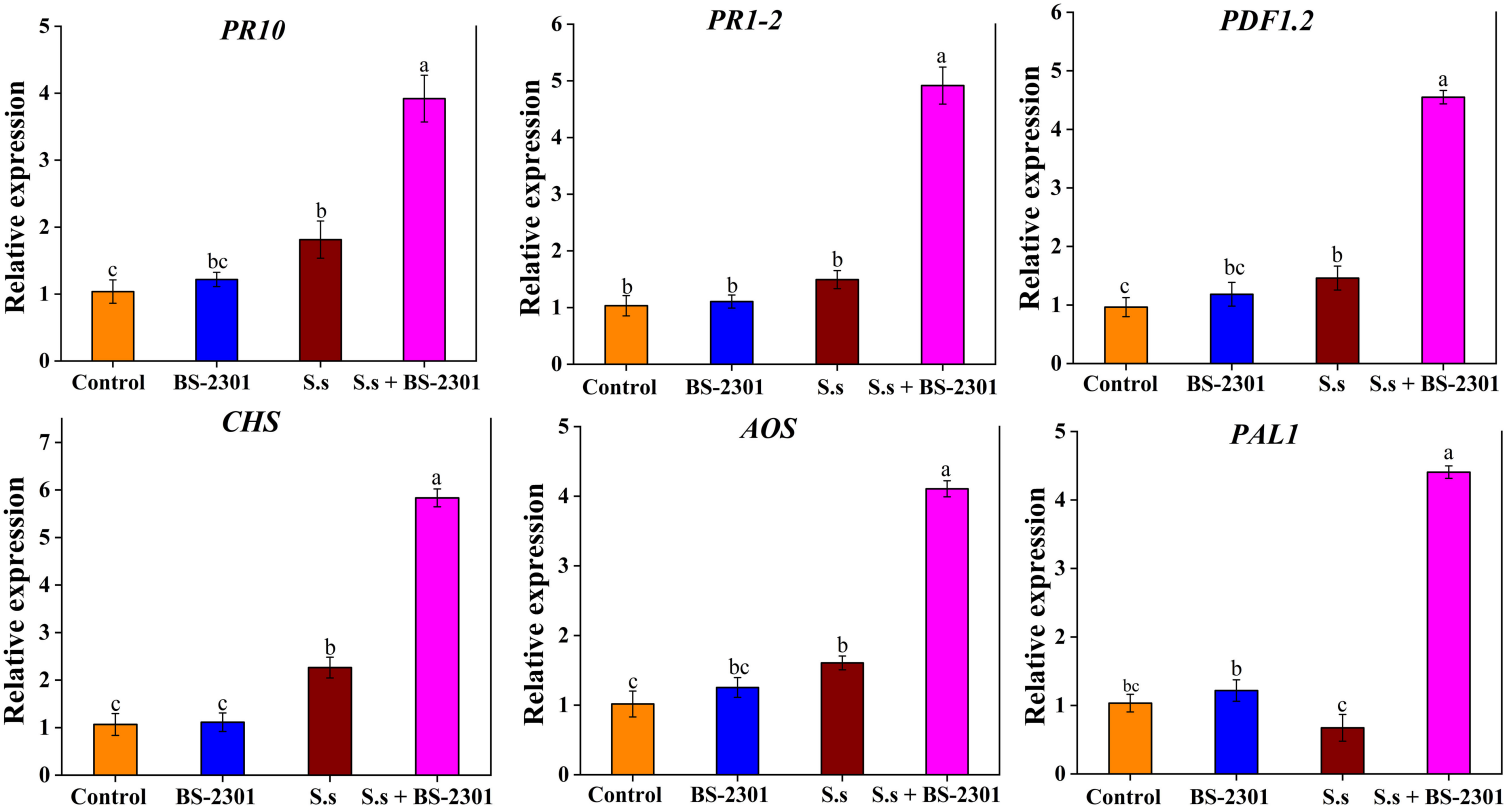
Expression profiling of six defense-related genes (*PR1-2*, *PR10*, *CHS*, *AOS*, *PAL1*, and *PDF1.2*) in soybean under different treatments. The significant difference among treatments was indicated by lowercase letters above the columns through Tuckey’s HSD test at P ≤ 0.05.

Soybean disease resistance could be associated with six genes: *PR10*, *PR1-2*, *PDF1.2*, *CHS*, *AOS*, and *PAL1*. The results revealed a 3-5 fold up-regulation of these genes in soybean plants treated with BS-2301 under *S. sclerotiorum* infection (S.s + BS-2301) compared to infected plants without BS-2301 (S.s) (**Figure 11**). The relative expression data indicated a significant upregulation of defense genes in infected soybeans plants under BS-2301 (S.s + BS-2301) compared to the S.s group. These results suggest that *B. subtilis* BS-2301 can enhance soybean resistance to *S. sclerotiorum* by triggering signaling pathways associated with PR proteins, plant defensins, and secondary metabolites.

## 4 Discussion

The safe and eco-friendly nature of biocontrol for plant diseases has sparked interest among researchers in studying beneficial microbes from various regions. *Bacillus* species are considered highly effective biocontrol agents of various phytopathogens (Farzand et al., 2019b; Ayaz et al., 2022). *Bacillus* strains exhibit numerous plant growth-promoting traits and suppress plant pathogens through various mechanisms. Previous research has identified several biocontrol agents for managing white stem mold disease in rapeseed plants (Chen et al., 2014; Ribeiro et al., 2021). The current study aimed to explore plant growth-promoting traits and biocontrol potential of the newly isolated strain BS-2301 against *S. sclerotiorum* in soybean plants. BS-2301 was identified as a *B. subtilis* strain based on 16S rRNA and whole genome sequencing. Previous studies have shown that *B. subtilis* inhibits a wide range of phytopathogens under laboratory conditions (Zhu et al., 2020). In the current study, BS-2301 exhibited broad-spectrum biocontrol efficacy against *S. sclerotiorum* (75%), *P. sojae* (72%), and *F. oxysporum* (70%) in dual culture and partition plate experiments. The direct inhibitory effect of BS-2301 and its crude extract was strongest against *S. sclerotiorum*, followed by *P. sojae* and *F. oxysporum*. Additionally, BS-2301 volatiles showed an indirect suppressive effect on the pathogens in partition plate experiments. Overall, BS-2301 exhibited strong biocontrol potential against *S. sclerotiorum* with a maximum growth inhibition rate of up to 74% compared to the control.

This study provides new insight into suppressing *S. sclerotiorum* through various mechanisms under BS-2301 treatments. The *S. sclerotiorum* hyphae were challenged with severe oxidative stress by exposure to BS-2301 or crude extract in direct contact or indirectly through BS-2301-VOCs. The results align with previous studies showing high ROS production in fungal mycelia exposed to biocontrol agents (Massawe et al., 2018; Ali et al., 2023). Our findings are consistent with (Massawe et al., 2018) who established a link between severe oxidative stress with negative effects on fungal cellular components, including DNA. BS-2301 induced severe oxidative stress in *S. sclerotiorum* like extreme temperatures, heavy metal stress, and UV radiation, leading to ROS accumulation surpassing the organism’s antioxidant defense capacity. The morphological and ultrastructural changes in treated *S. sclerotiorum* observed under TEM and SEM microscopy match the findings from previous studies, including breakdown of cell membranes, cell breakage, plasmolysis, cytoplasm displacement, and organelle disintegration (Giorgio et al., 2015; Ntalli et al., 2017). Oxalic acid (OA) production is a key factor in *S. sclerotiorum* pathogenicity, facilitating the creation of a conducive environment for hydrolytic enzymes (Massawe et al., 2018; Farzand et al., 2019a). Our study hypothesized that BS-2301 could affect OA production in *S. sclerotiorum*. The bromophenol blue assay showed reduced acidification, indicating lower OA production in *S. sclerotiorum* challenged with BS-2301 and crude extract than in the control. The *Bacillus* VOCs have been demonstrated to modify *S. sclerotiorum* OA in reduced pathogenicity, suggesting that decreased production of OA may weaken the host-pathogen interaction (Massawe et al., 2018; Zhu et al., 2022). Furthermore, BS-2301 markedly reduced sclerotia development, which is a resilient structure that can survive harsh conditions for years before germinating into infective hyphae (Hegedus and Rimmer, 2005; Ordóñez-Valencia et al., 2015). The interference of BS-2301 with fungal mycelia likely contributed to the reduction in sclerotia formation. The studies by (Kai et al., 2009) and (Massawe et al., 2018) have shown that *Bacillus* species VOCs can inhibit sclerotia germination. Our findings suggest that BS-2301 and its crude extract may disrupt sclerotia melanin synthesis by interacting with tyrosin oxidation products and dehydroxyphenol compounds, the key components of melanin found in sclerotia.

The PGPR has been shown to support plant growth in harsh conditions through extracellular enzymes, siderophores and IAA production (Zubair et al., 2021; Ali et al., 2022). *Bacillus* spp. produce enzymes like chitinase, protease, β-1,3-glucanase, and cellulase (Ajuna et al., 2023). Researchers have extensively studied *Bacillus* due to its remarkable characteristics (Patel et al., 2023). For example, *B. cereus* YN917 produces various enzymes and exhibits mineral phosphate decomposition activity (Zhou et al., 2021). *Bacillus velezensis* NKG-2 has been shown to produce cellulase, β-glucanase, chitinase, IAA, and siderophore (Myo et al., 2019). The *B. subtilis* BS-2301 in the present study produced extracellular enzymes such as amylase, cellulases, and proteases and exhibited PGP traits such as siderophores production, IAA synthesis, and phosphorus-solubilizing activity. These traits may help soybean plants survive infections with *S. sclerotiorum*. Additionally, different gene clusters in BS-2301 genome were predicted for important antimicrobial compounds like bacilysin, fengycin, and surfactant. Previous studies have demonstrated the remarkable potential of fengycin produced from *B. amyloliquefaciens* FZB42 in managing *S. sclerotiorum*. It was found that fengycin triggered ISR in infected tomato plants and downregulated the pathogenicity genes in *S. sclerotiorum* (Farzand et al., 2019a). Surfactin is an important antimicrobial lipopeptide with antifungal activities against multiple plant pathogenic fungi and might have potential uses in agriculture. The surfactin isolated from *Brevibacillus brevis* KN8(2) has been evaluated for its antifungal activity against *F. moniliforme*. The study suggested that surfactin might be an effective bio-fungicide for controlling plant diseases (Krishnan et al., 2019). Previous studies showed bacilysin an effective biocontrol agent for *Xanthomonas* disease suppression in rice plant. The expression of genes related to *Xanthomonas* pathogenicity, cell division and cell wall synthesis was downregulated under bacilysin treatment (Wu et al., 2015). In present study, the BS-2301 genome was also found to possessed surfactin, fengycin and bacilysin gene clusters that might suppress *S. sclerotiorum* through various mechanisms.

The scientific community has shown considerable interest in identifying novel beneficial microbes to control phytopathogens and improve plant health (Ayaz et al., 2023). The study by (Khan et al., 2020) reported *B. subtills* KSU-110 reduced *Fusarium* wilt disease of tomato in greenhouse experiments. In present studies, BS-2301 was found to make soybean resistant to *S. sclerotiorum* under pot experiment. Our study revealed that soybean leaves in BS-2301 treated plants exhibited enhanced activity of POD, SOD, CAT, and APX compared to the control. It was previously reported that PGPR in host plants regulates antioxidant enzymes like POD, SOD, and CAT which help in removing excessive ROS and preventing pathogen invasion (Ali et al., 2023; Zhai et al., 2023). Our results showed up-regulation of antioxidant enzymes in infected soybean plants, possibly involved in reducing excessive ROS production after *S. sclerotiorum* infection. The active antioxidant enzyme system plays a crucial role in maintaining normal plant cell growth. Pathogen and abiotic stressors can damage plant cells, leading to MDA production, and indicating cell death or degeneration (Liu et al., 2021). Soybean treated with the BS-2301 strain had lower MDA content compared to *S. sclerotiorum*-infected soybeans, suggesting reduced cellular damage in treated plants. This indicates that BS-2301 can trigger defense-related enzyme activities in soybeans, reducing *S. sclerotiorum* infection. our findings support previous research showing up-regulation of antioxidant enzymes and reduced MDA content in rice infected with *Xanthomonas oryzae* and *Ralstonia solani* under *Bacillus* VOCs treatment (Ali et al., 2023). Moreover, PGPR may trigger ISR by interacting with plant defense genes and signaling pathways to minimize plant pathogen infection (Salwan et al., 2023). Gene expression profiling revealed increased expression of defense-related genes (*PR1-2, PR10, PDF1.2, CHS, PAL1*, and *AOS*) in soybean plants treated with BS-2301, indicating significant up-regulation of plant defense genes. Our results align with previous research showing that *Klebsiella variicola* FH-1 strain activated ISR against *S. sclerotiorum* in soybeans by positively regulating defense-related genes (Zhai et al., 2023). In conclusion, our study demonstrates that the newly isolated *B. subtilis* BS-2301 exhibits a plethora of plant growth-promoting traits and biocontrol potential to suppress different plant pathogens, trigger ISR, reduce stem lesions, enhance antioxidant enzymes, and regulate plant defense genes to control *S. sclerotiorum* disease in soybean plants.

## 5 Conclusion

The present study identified a newly isolated *B. subtilis* BS-2301 with potent broad-spectrum biocontrol potential against *S. sclerotiorum*, *P. sojae*, and *F. oxysporum*. The findings shed light on BS-2301 biocontrol mechanism against *S. sclerotiorum* involving excessive ROS production leading to pathogen cell death. The BS-2301 induced morphological and ultrastructural changes in *S. sclerotiorum* mycelia, including cell wall and membrane breakage, cytoplasmic displacement, and organelle disintegration. Treatment with BS-2301 resulted in reduced oxalic acid production and sclerotia formation which are considered key pathogenicity factors in *S. sclerotiorum* infection. Additionally, BS-2301 promoted soybean plant growth and inhibited *S. sclerotiorum* disease progression on soybean leaves and stem in both *in vitro* and pot experiments. The plant-growth-promoting and biocontrol efficacy of BS-2301 may be attributed to extracellular enzymes, secondary metabolites, VOCs, IAA synthesis, and stable biofilm formation. BS-2301 induced systemic resistance in soybean plants by regulating antioxidant enzymes and defense-related genes during *S. sclerotiorum* infection. Overall, *B. subtilis* BS-2301 exhibited plant growth-promoting potential and effectively suppressed *S. sclerotiorum* through various mechanisms. This study lays the foundation for managing *S. sclerotiorum* disease in soybean plants, with further research need to evaluate BS-2301 efficacy in field trials.

## Data Availability

The original contributions presented in the study are included in the article/**Supplementary Material**. Further inquiries can be directed to the corresponding authors.
