# Supplementary material for: Exploring plant growth promoting traits and biocontrol potential of new isolated Bacillus subtilis BS-2301 strain in suppressing Sclerotinia sclerotiorum through various mechanisms
Source: Front Plant Sci. 2024 Aug 22;15:1444328. doi: 10.3389/fpls.2024.1444328 (PMC11374654; doi:10.3389/fpls.2024.1444328)
Supplement: Supplementary file 1 [file Table1.docx]

| Defense related genes | | |
| --- | --- | --- |
| *PR-1-2-F* | Forward Primer | GTGGTGAATACGGAGAG |
| *PR-1-2-R* | Reverse Primer | CTCCTCCAACACAAGAG |
|  |  |  |
| *PR-10-F* | Forward Primer | GCTCCTGCTACTCTTTAC |
| *PR-10-R* | Reverse Primer | CCACCATTTCCCTCAAC |
|  |  |  |
| *PDF1.2* | Forward Primer | CCTCCTCCTTGTCTTTG |
| *PDF1.2* | Reverse Primer | GGATTCCAATTCCTCCTC |
| *PAL-1-F* | Forward Primer | GGAGTCTCTATGGACAAC |
| *PAL-1-R* | Reverse Primer | CCCTTGAACCCATAGTC |
|  |  |  |
| *AOS-F* | Forward Primer | GTCCTCTTCGACAACTC |
| *AOS-R* | Reverse Primer | GGGTCGAGGTAAGAAAG |
|  |  |  |
| *CHS-F* | Forward Primer | CCCATCTTGATAGCCTTG |
| *CHS-R* | Reverse Primer | CACTGTCTGGAAGGATTG |
|  |  |  |
| *Actin-F* | Forward Primer | GTTATGGTTGGGATGGG |
| *Actin-R* | Reverse Primer | CCCAGTTGCTGACTATAC |
